# The epidemiology of tuberculosis in health care workers in South Africa: a systematic review

**DOI:** 10.1186/s12913-016-1601-5

**Published:** 2016-08-20

**Authors:** Liesl Grobler, Shaheen Mehtar, Keertan Dheda, Shahieda Adams, Sanni Babatunde, Martie van der Walt, Muhammad Osman

**Affiliations:** 1 Center for Evidence-based Health Care, Stellenbosch University, Cape Town, South Africa; 2 Unit of Infection Prevention and Control, Stellenbosch University, Cape Town, South Africa; 3 Lung Infection and Immunity Unit, Division of Pulmonology and University of Cape Town Lung Institute, Department of Medicine, University of Cape Town, Cape Town, South Africa; 4 Centre for Occupational and Environmental Health Research, University of Cape Town, Cape Town, South Africa; 5 World Health Organisation, Pretoria, South Africa; 6 Tuberculosis Research Platform, South African Medical Research Council, Pretoria, South Africa; 7 Cape Town City Health Department, Cape Town, South Africa

**Keywords:** Tuberculosis, Health care workers, Health personnel, Sub-Saharan Africa

## Abstract

**Background:**

In South Africa, workplace acquired tuberculosis (TB) is a significant occupational problem among health care workers. In order to manage the problem effectively it is important to know the burden of TB in health care workers. This systematic review describes the epidemiology of TB in South African health care workers.

**Methods:**

A comprehensive search of electronic databases [MEDLINE, EMBASE, Web of Science (Social Sciences Citation Index/Science Citation Index), Cochrane Library (including CENTRAL register of Controlled Trials), CINAHL and WHO International Clinical Trials Registry Platform (ICTRP)] was conducted up to April 2015 for studies reporting on any aspect of TB epidemiology in health care workers in South Africa.

**Results:**

Of the 16 studies included in the review, ten studies reported on incidence of active TB disease in health care workers, two report on the prevalence of active TB disease, two report on the incidence of latent TB infection, three report on the prevalence of latent TB infection and four studies report on the number of TB cases in health care workers in various health care facilities in South Africa. Five studies provide information on risk factors for TB in health care workers. All of the included studies were conducted in publicly funded health care facilities; predominately located in KwaZulu-Natal and Western Cape provinces. The majority of the studies reflect a higher incidence and prevalence of active TB disease in health care workers, including drug-resistant TB, compared to the surrounding community or general population.

**Conclusions:**

There is relatively little research on the epidemiology of TB in health care workers in South Africa, despite the importance of the issue. To determine the true extent of the TB epidemic in health care workers, regular screening for TB disease should be conducted on all health care workers in all health care facilities, but future research is required to investigate the optimal approach to TB screening in health care workers in South Africa. The evidence base shows a high burden of both active and latent TB in health care workers in South Africa necessitating an urgent need to improve existing TB infection, prevention and control measures in South African health care facilities.

**Electronic supplementary material:**

The online version of this article (doi:10.1186/s12913-016-1601-5) contains supplementary material, which is available to authorized users.

## Background

Health care workers have an increased risk of acquiring tuberculosis (TB) as they are exposed to TB in their community as well as at their place of work [1–3]. In South Africa, workplace acquired TB is an important occupational disease among health care workers. According to the 2006 compensation fund claims, tuberculosis in health care workers, whilst generally underreported, is the third most commonly reported occupational disease in South Africa [4]. A review of the data submitted to the Compensation Commissioner by health care workers regarding hospital acquired infections from January 2007 to December 2009 in Limpopo province (i.e. one of the nine administrative divisions within the country) found that TB was the most common hospital acquired infection, with 47 (83.9 %) of the 56 reported cases of infectious diseases being TB cases [5].

Furthermore, the Human Immunodeficiency Virus (HIV) epidemic has impacted greatly on TB in South African health care workers. The HIV epidemic has increased the patient burden with TB co-infection in health care facilities, particularly at the primary care level. The increased number of sick patients with HIV-related TB has increased health care workers’ exposure to occupationally acquired diseases such as TB. Additionally, 11–20 % of South African health care workers are themselves, HIV-positive [6–8]. These HIV-infected health care workers have an increased risk for acquiring TB as well as for progressing from latent TB to active clinical disease [6].

Despite international policy recommendations [9] and national legislative provisions to address workplace acquired TB, generally South African health care facilities do not have adequate or appropriate infection prevention and control measures in place to protect their employees [10] (Dwadwa et al: Health worker access to HIV/TB prevention, treatment and care services in Africa: situational analysis and mapping of routine and current best practices, unpublished). The poorly enforced TB infection prevention and control policies (including administrative, engineering and personal protective equipment measures), the over-crowded health care facilities with insufficient ventilation to allow for appropriate environmental infection control and the high levels of undiagnosed, infectious TB patients, all contribute to health care workers’ increased risk of exposure to TB in South African health care facilities.

The success of any TB Control Programme is dependent on health care workers’ knowledge and application of appropriate policies and practices. In 2010 there were only 3.97 medical practitioner per 10 000 population and 18.97 professional nurses per 10 000 population in South Africa [11], reflecting the scarcity of health care workers in an overburdened health care system. TB infection amongst health care workers results in absenteeism and sometimes death or disability of health care workers, further weakening the already overburdened health care system.

The first step towards managing workplace acquired TB in South African health care workers is to determine the true burden of TB in this population. This systematic review aims to collate all research conducted in South Africa reporting on any aspect of epidemiology of TB in health care workers in South Africa.

## Methods

This systematic review is part of a larger project aimed at identifying all scientific evidence on the epidemiology of, and programmatic response to, TB in South African health care workers (http://www.cebhc.co.za/research-key-outputs/research-evisat/). The larger project collated information on TB epidemiology, TB infection, prevention and control and TB prevention, treatment and care among health care workers in South Africa. Only the results of the epidemiology of TB in health care workers will be presented here.

### Criteria for studies for inclusion in this review

Based on previously published definitions, the term “health care worker” was defined as “all people engaged in actions whose primary intent is to enhance health” and included, but was not restricted to, physicians, nurses, allied health personnel, health educators, social workers, midwives, community health workers, laboratory personnel, pharmacists, radiographers, volunteers, orderlies, and health-facility administrators [2, 12]. All studies reporting on any aspect of TB epidemiology in health care workers in South Africa were presented in this review.

### Search methods for identification of studies

A comprehensive search strategy (see Table 1), without any date or language restrictions, was used to search the following databases:

- MEDLINE (1966 to April 2015)
- EMBASE (1947 to April 2015)
- WEB OF SCIENCE (Social Sciences Citation index/Science Citation Index—1970 to April 2015)
- Cochrane Library (up to Issue 5 2013)—including CENTRAL register of Controlled Trials
- CINAHL (1981 to April 2015)
- WHO International Clinical Trials Registry Platform (ICTRP) up to April 2015

**Table 1:** Detailed search strategy

Search terms for MEDLINE (1966 to April 2015) 1. South Africa [Mesh], ti, ab 2. Tuberculosis [Mesh], ti, ab 3. 1 and 2 4. “healthcare worker*” OR “health care worker*” OR nurse* [ti, ab] OR “Health Personnel” [Mesh] 5. 3 and 4 The strategy was amended where necessary to search the other databases listed below: − EMBASE (1947 to April 2015) − WEB OF SCIENCE (Social Sciences Citation index/Science Citation Index—1970 to April 2015) − Cochrane Library (up to Issue 5 2013)—including CENTRAL register of Controlled Trials − CINAHL (1981 to April 2015) − WHO International Clinical Trials Registry Platform (ICTRP) up to April 2015
Following feedback from stakeholders at the EVISAT workshop an additional search for relevant information was conducted using the following search terms: 1. “Infection control” [Mesh] 2. “South Africa” 3. #1 AND #2 The records retrieved from this search were screened in the same way as the initial search to see if any additional relevant references were retrieved.

To identify additional research we contacted experts (for example, researchers, health care workers, South African Department of Health employees) working in the field of TB in South Africa and screened the reference lists of eligible studies and review articles.

### Data collection and analysis

Three reviewers independently screened the results of the searches (Liesl Nicol, Shaheen Mehtar and Keertan Dheda) and extracted data (Liesl Nicol, Muhammad Osman and Lydia Mudzikati) from eligible studies using a standardised data extraction form. Any disagreements regarding study eligibility or data extraction were resolved by discussion among the review team. The degree of selection bias, detection/information bias and confounding was determined for each included study using a validated risk of bias tool [13]. We planned to summarise the incidence data on TB in health care workers. However, after extracting the data from the eligible studies we noted that only some of the studies reported the 95 % confidence interval or standard deviation around the mean incidence or prevalence values. After consultation with a statistician it was agreed that the high level of heterogeneity between the incidence values would make summation of the data meaningless. Therefore, we decided to present the data in a horizontal bar chart to graphically reflect the incidence of active TB disease in health care workers compared to the general population or surrounding community.

## Results

### Search results

**Fig. 1:**
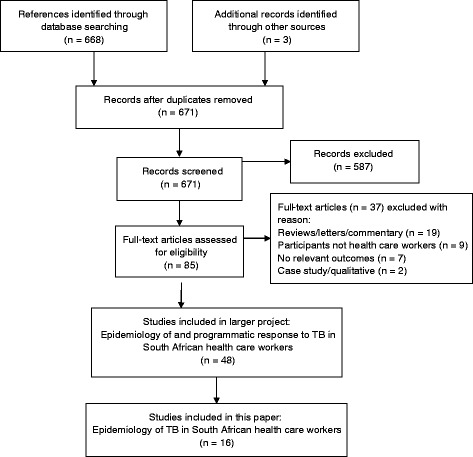
Flow diagram showing selection of studies

The electronic search yielded a total of 668 references and searching other resources (for example: screening references of included studies, contacting experts in the field) yielded three additional studies (Fig. 1). A total of 587 references were excluded after the initial screening as the references either did not pertain to health care workers or the studies were not conducted in South Africa. We obtained the full text articles of 85 references, 48 of which were eligible for inclusion in the larger review looking at the epidemiology of and programmatic response to TB in South African health care workers. The reasons for excluding the remaining 37 references are outlined in Additional file 1: Table S1. The data from studies reporting on TB infection prevention and control, TB occupational health and safety, and care of health care workers with TB will be summarised in a future paper.

### Description of included studies

We identified 16 studies reporting on various aspects of TB epidemiology in health care workers in South Africa. The detailed characteristics and findings of these studies are presented in Table 2.

**Table 2:** Incidence and prevalence of active TB disease and latent TB infection in health care workers in South Africa

Study ID	Study characteristics	Study outcomes	General comments
National			
Claassens et al. [15] (URC report)	Study date: 2009 133 primary health care facilities in Limpopo, Eastern Cape, Kwazulu-Natal, Mpumalanga and North-West provinces. No information provided on the health care workers	Incidence of active TB disease (smear positive) 2006: 834 (95%CI 431–1457) per 100,000 persons 2007: 1092 (95%CI 647–1725) per 100,000 persons 2008: 887 (95%CI 517–1420) per 100,000 persons	To compare the TB incidence rate among healthcare workers with the general population the incidence rate was combined for all facilities. For comparison with the general population a standardised incidence ratio was calculated. The standardised incidence ratio for smear positive TB in healthcare workers in: 2006: 2.4 (95%CI 1.2–4.2) 2007: 3.0 (95%CI 1.8–4.7) 2008: 2.3 (95%CI 1.3–3.7)
Dwadwa et al: Health worker access to HIV/TB prevention, treatment and care services in Africa: situational analysis and mapping of routine and current best practices, unpublished Situational analysis	Study date: 2006 Publicly funded health care facilities in KZN, Limpopo, North West, Northern Cape and Western Cape Number of participants: 173 health care workers from 10 facilities Age: Not reported Sex: Not reported HIV status: Not reported Method of TB diagnosis: Unclear	Incidence of active TB disease 2006: Between 1130 and 1470/100,000 staff had been treated for TB during 2006.	In 2006 the national TB case notification rates of South Africa were 719.9 per 100,000.
KwaZulu-Natal			
Wilkinson et al. [21]	Study date: not reported Study setting: A district hospital in rural KwaZulu-Natal Number of participants: 725 total number of staff at risk 22 TB cases from 1991 to 1996 Age: Mean age of staff at risk = 37 years; Mean age of TB cases = 29.6 years Sex: 36 % of staff at risk was female. 64 % of TB cases were female HIV status: 59 % of the staff at risk who were tested for HIV were HIV+ 54.5 % of TB cases who were tested for HIV were HIV+ Method of diagnosing TB: Smear microscopy, chest X-RAY	Annual incidence of active TB disease 138/100 000 (1991/1992) Annual incidence of active TB disease 690/100 000 (1993–1996)	1991–1996: Annual incidence of TB in the surrounding community was 1543/100 000. Mean age of affected health care workers was 29 years No significant difference in incidence of TB disease between occupational categories Of the 14 HCWs who were tested for HIV, 12 were HIV-positive
Naidoo et al. [16]	Study date: 2004/2005 Study setting: Eight public sector hospitals in Ethekwini municipality, KwaZulu-Natal Number of participants: 583 cases of HCWs with TB during the study period Age: HCWs with TB: mean = 37.9 years (range: 21–64) Sex: HCWs with TB: 64 % female HIV status: Not reported Method of diagnosing TB: Not reported	Mean incidence of active TB disease 1133/100 000 (standard deviation: 282.8, 1999–2004)	Mean incidence of TB disease among general population in KZN over study period: 497/100 000 Highest incidence of TB disease in 25–29 year olds (2468/100 000; *p* = 0.003) and paramedical staff (1558/100 000; not statistically significantly different to the incidence in the other occupational categories).
O’Donnell et al. [17]	A public TB referral hospital in KwaZulu-Natal Number of participants: 4941 total admissions of M(X) DR-TB 231 confirmed HCWs (MDR-TB = 208; XDR-TB = 23) Age: Median = 35 years Sex: 78 % female HIV status: 55 % of HCW with M(X) DR-TB were HIV+ Method of diagnosing TB: Culture confirmed TB with MTB drug susceptibility testing	Annual estimated incidence of MDR-TB hospital admissions among health care workers (2003–2008) 64.8/100 000 Annual estimated incidence of XDR-TB hospital admissions among health care workers (2003–2008) 7.2/100 000	Annual incidence of MDR-TB hospital admissions among the adult general population in KwaZulu-Natal: 11.9/100 000 (I.R.R. 5.56; 95 % CI: 4.86–6.35). Annual incidence of XDR-TB hospital admissions among adult general population in KwaZulu-Natal: 1.1/100 000 (I.R.R. 6.69; 95 % CI: 4.38–10.2)
Tudor et al. [18]	Study date: 2011 Three district hospitals in KwaZulu-Natal, South Africa, with specialized drug-resistant tuberculosis (TB) wards Number of participants: 1427 HCW occupational health records Age: Median = 39 years Sex: 78.05 % female HIV status: 24 % of HCWs with TB HIV+ Method of diagnosing TB: Not reported	Incidence of active TB disease 1958/100 000 (2010) 2006–2010: 112 (8.53 %) cases of TB, of which 14 (12.5 %) cases were drug-resistant TB.	Incidence of TB disease among the general population of KwaZulu-Natal was 1142/100 000 (2011). Throughout the study period health care workers had significantly higher annual TB incidence rate ratios (IRR) for each year of the study compared to that of the general population in KwaZulu-Natal. Incidence of active TB in South Africa: 981/100 000 (2010)
Western Cape			
Adams: Prevalence and determinants of TB infection in health care workers, unpublished	Study date: 2009–2011 7 primary and secondary level health care facilities in the Western Cape province Number of participants: 505 Age: <30 years (25 %); 31–40 years (27 %); 41–50 years (27 %); >50 years (22 %) Sex: 74 % female HIV status: Primary level staff 19 % HIV+; Secondary level staff 2 % HIV+ Method of diagnosing TB: TB infection: TST, QFT-GIT, TSPOT.TB. TB disease: TB symptom screen, Chest X-Ray and 2× sputum culture.	Annual incidence of active TB disease 900/100 000; 95 % CI: 0.2–2.6 (2009–2011) Prevalence of active TB disease 1400/100 000 (2009–2011) Prevalence of latent TB infection 84 % (TST; 2009–2011) Annual incidence of latent TB infection 38 % of HCWs converted from TST negative to positive (2009–20110)	Annual incidence rate in Cape Town 799/100 000 population and Western Cape 935/100 000 population (2008–2011) 3/5 HCWs with active TB were HIV+ 9 % of HCWs reported being diagnosed with or on treatment for diabetes Annual incidence of latent TB infection QFT-GIT conversion rate 22 % reversion rate 7 % T-SPOT.TB conversion rate 22 % reversion rate 16 %
Ayuk et al. [20]	Study date: 2008–2011 Tygerberg Academic Hospital, Western Cape province Number of participants: 249 Age: Mean = 43.8 (range: 23–60) years Sex: 71.4 % female HIV status: 30 % HIV+; 63 % HIV-; 6 % unknown Method of diagnosing TB: smear+; culture+, histology and symptoms	Mean annual incidence rate of active TB disease 2008–2011: 397/100 000 population (95%CI: 307–505 per 100 000; all health care workers) Incidence rate of active TB disease Housekeeping staff: 1181/100 000 Nurses: 324/100 000 Doctors: 194/100 000	Annual incidence rate in Cape Town 799/100 000 population and Western Cape 935/100 000 population Housekeeping staff incidence rate of active TB disease approximately 3 times (95%CI: 2.7–3.3) that of the entire workforce, 3.6 times (95%CI: 3.2–4.1) that of nurses and 6.1 times (95%CI: 5.2–7.1) that of doctors.
Kranzer et al. [6]	Study date: 2008/2009 Mobile HIV testing unit (the TUTU tester) provided HIV testing, CD4 counts and TB screening to TB and antiretroviral adherence supporters employed by the TB/HIV Care Association in Cape Town on 8 days in 8 venues in the Western Cape province Number of participants: 215 community health workers (CHW) were offered HIV and TB testing Age: Most common age group was 40–49 years old (*N* = 72, 33 %) Sex: All female HIV status: 42 CHW HIV+ (20 %) Method of diagnosing TB: Smear and culture testing	Prevalence of active TB disease 5 % (10/215; 2008–2009)	
Naidoo et al. [24]	Date of study not reported Western Cape Number of participants: 100 randomly selected practicing dentists; 78 % response rate. Age: Mean = 40 years Sex: 80 % male HIV status: Not reported Method of diagnosing TB: Mantoux and multipuncture tests	Prevalence of latent TB infection 33 % (No dates provided)	
Mehtar et al. [29]	Study date: 2010 Retrospective review of OATB case reports at Tygerberg Academic Hospital in the Western Cape Number of participants: 67 cases of OATB reported during study period 2008–2010 Age: Not reported Sex: Not reported HIV status: Not reported Method of TB diagnosis: Smear microscopy	Number of occupationally acquired TB cases 2008: 27 TB cases (17 ancilliary staff) 2009: 21 TB cases (7 ancilliary staff) 2010: 19 TB cases (13 ancilliary staff) Pulmonary TB among health care workers 38.5 % (2008) 27.8 % (2010)	
Jarand et al. [25]	Study date: 1996–2008 Retrospective case record review of patients with XDR-TB from Eastern and Western Cape provinces Number of participants: 334 XDR-TB patients (10 HCWs with XDR-TB) Median age: 41 (26–50) Sex: 90 % female HIV status: 2/10 HIV+ Method of TB diagnosis: sputum culture	10 of the 334 patients with diagnosed with XDR-TB between 1996 and 2008 were health care workers	5/10 were nurses 4/10 HCWs died HCW with XDR TB: 26–50 years old; 60 % nursing staff; 80 % HIV-negative
Gauteng			
McCarthy et al. [22] Van Rie et al. [23]	Study date: 2008–2009 Gauteng Number of participants: 79 medical students and 120 healthcare workers Age: Medical students median age 22 (22–24) HCW median age 36 (28–46) Sex: Medical students = 44 % males HCW = 10 % males HIV status: Medical students = 0 % HIV+; HCW = 18.3 % HIV+ Method of diagnosing latent TB: TST and IGRA assay (Quantiferon-TB Gold In-tube)	Incidence of latent TB infection (2008–2009) 26 % (29 per 100 personyears, 95%CI: 20–44, IGRA) 27 % (29 per 100 person years, 95%CI: 19–42, TST) Prevalence of latent TB infection (2008) 45 % (95%CI: 38–53; IGRA+) 48 % (95%CI: 42–57; TST+) Incidence of active TB disease (2008–2009) 1.8/100 person years (95%CI: 0.45–7.2) among HCWs	HCWs LTBI prevalence was two-to four-fold higher than medical students TST: 56.7 % vs. 26.6 % IGRA: 69.2 % vs. 15.2 %
Limpopo			
Malangu et al. [5]	Study date: not reported Data on health care acquired infection submitted to the Compensation Commissioner of the Limpopo province by a health care worker Number of participants: 56 cases of infectious diseases reported over 2 years. Age: 42.7 years (mean) Sex: 67.9 % female HIV status: Not reported Method of TB diagnosis: Unclear	2007–2009: Of the 56 reported cases of infectious diseases 47 (83.9 %) were TB cases. 30/47 TB cases were nurses 8/47 TB cases were cleaning staff	Among health professionals, nurses most likely to acquire TB disease; Among non-health professionals cleaning staff most likely to acquired TB disease Cases were defined as reports about any healthcare acquired infection as submitted for compensation by healthcare workers of Limpopo province.
Mpumalanga			
Balt et al. [26]	Study date: Not reported Study setting: 4 dedicated TB centres (SANTA centres) in Mpumalanga Details of study participants and method of TB diagnosis not reported	Annual incidence of TB disease (1986–1997) 275/100 000 (95 % confidence interval: 33–991) Between 1986 and 1997: 2 nurses with active TB	The incidence of TB in the general population of Mpumalanga at the time estimated to be 286/100 000 Affected nurses were 36 and 56 years old; both nurses had non-insulin dependant diabetes

#### Study setting and location

All of the included studies were conducted in publicly funded (government-funded), as opposed to privately funded, health care facilities in South Africa. Studies were conducted in health care facilities located in KwaZulu-Natal and the Western Cape, two of the nine South African provinces (i.e. administrative divisions within the country). We did not find any studies reporting on TB epidemiology in health care workers in the Eastern Cape, Free State, the Northern Cape and the North West province. The included studies provide information on TB epidemiology in health care workers in primary, secondary and tertiary level health care facilities. One study was conducted from a mobile testing unit in a community-based, as opposed to facility-based, setting [6].

#### Study design and risk of bias

Eight retrospective cohort studies, six cross-sectional studies and two prospective cohort study provided information on the epidemiology of TB in health care workers. Table 3 provides a summary of the risk for selection bias, detection/information bias and confounding present in each of the included studies. The risk of selection bias and detection/information bias was judged to be low in the retrospective cohort studies as these studies extracted data from hospital records or databases using data extraction forms. However, it is well known that there is underreporting of TB cases among health care workers, which may introduce detection/information bias and affect the external validity of the studies. Confounding was judged to be unclear in most of the studies as it is often unclear if the TB was acquired in the community or in the work place because of the high background incidence and prevalence of TB in South Africa. This issue is likely to confound most studies in this setting (Table 3).

**Table 3:** Risk of bias assessment for studies reporting on incidence and prevalence of active TB disease and latent TB infection in health care workers

Study ID	Risk of selection bias^a^	Risk of detection/information bias for each outcome^b^	Risk of confounding^c^
Prospective cohort study			
Adams: Prevalence and determinants of TB infection in health care workers, unpublished	UNCLEAR: Participation in the study was voluntary. 505/764 HCWs participated in the study.	Incidence and prevalence of LTBI and TB disease: LOW (TB diagnosed using standard procedures) Risk factors for TB: HIGH (HCWs completed questionnaires which are prone to information bias)	UNCLEAR: Community vs. occupational exposure to TB; HIV status of all of the HCWs not known
McCarthy et al. [22] (linked to Van Rie et al. [23])	UNCLEAR: Participation in the study was voluntary. 120/450 eligible HCWs and 79/296 eligible medical students participated in the study.	Incidence and prevalence of LTBI: LOW (latent TB infection diagnosed using standard procedures) Risk factors for TB: HIGH (HCWs completed questionnaires which are prone to information bias)	UNCLEAR: Community vs. occupational exposure to TB. However, HIV status of all participants was assessed.
Cross sectional study			
Ayuk et al. [20]	UNCLEAR: Not all HCWs completed questionnaire	Incidence and prevalence of TB disease: LOW Risk factors for TB: HIGH (HCWs completed questionnaires which are prone to information bias)	UNCLEAR: Community vs. occupational exposure to TB; HIV status of all of the HCWs not known
Claassens et al. [15]	LOW: Although authors report that health care facilities were randomly selected there is no explanation of how the randomisation process was conducted.	Incidence of TB disease: HIGH (In each health care facility a questionnaire was completed by the facility manager to indicate the number of HCWs who were registered in that facility and who had been on TB treatment from January 2006 to December 2008.	UNCLEAR: Community vs. occupational exposure to TB; HIV status of all of the HCWs not known
Dwadwa et al: Health worker access to HIV/TB prevention, treatment and care services in Africa: situational analysis and mapping of routine and current best practices, unpublished	UNCLEAR: Six of the facilities were randomly selected although there is no explanation of how this was conducted. Four of the facilities were specifically selected based on current best practice as recommended by the Department of Health and AIDS and TB Directorates.	Number of TB cases: HIGH (Data obtained from questionnaires and interviews	UNCLEAR: Community vs. occupational exposure to TB
Kranzer et al. [6]	UNCLEAR: It is not clear how the community health workers (CHWs) were selected, if all of the CHW were selected to participate or if participation was voluntary	Prevalence of TB disease: LOW (standard TB diagnostic techniques used)	UNCLEAR: HIV status of all of the HCWs not known
Naidoo et al. [24]	UNCLEAR: Although authors state that a randomly selected sample of dentists was approached to participate in the study, it is not clear how this randomisation process was conducted. Only 78 of the 100 dentists participated	Prevalence of LTBI: LOW (LTBI diagnosed with Mantoux tests)	UNCLEAR: It is not clear where the dentists practiced or the demographics of their patients; Community vs occupational exposure to TB; HIV status of all of the HCWs not known
Retrospective cohort study			
Balt et al. [26]	LOW: Detailed review of health staff records at the four dedicated TB centres	Incidence of TB disease: LOW	UNCLEAR: Community vs. occupational exposure to TB
Malangu et al. [5]	LOW: A pre-designed data collection form was used to extract data from claims submitted to the Compensation Commissioner from January 2007 to December 2009	Cases of TB disease: LOW (Data based on reported cases of TB. However, it is well known that there is underreporting of TB among HCWs with regards to occupational diseases. This may introduce detection bias and affect the external validity of the study)	UNCLEAR: possible underreporting of TB cases; Community vs occupational exposure to TB; HIV status of all of the HCWs not known
Jarand et al. [25]	LOW: Retrospective case record review of all patients with XDR-TB in Eastern and Western Cape province from 1996 to 2008	UNCLEAR: it is not known how study authors determined whether patients were health care workers	UNCLEAR: Community vs. occupational exposure to TB
Mehtar et al. [29]	LOW: Retrospective review of occupationally acquired TB case reports	Cases of TB disease: LOW (Data based on reported cases of OATB. However, it is well known that there is underreporting of TB among HCWs with regards to occupational diseases. This may introduce detection bias and affect the external validity of the study)	UNCLEAR: Underreporting of TB cases; Community vs occupational exposure to TB; HIV status of all of the HCWs not known
Naidoo et al. [16]	LOW: Retrospective record review. All HCW with TB treated at 8 specified public sector hospitals were included if records confirmed HCW status	Incidence of TB disease: LOW (However, it is possible that HCWs seeking TB treatment may not have stated their occupation, resulting in underreporting of TB cases and information bias)	UNCLEAR: Underreporting of TB cases; Community vs occupational exposure to TB; HIV status of all of the HCWs not known
O’Donnell et al. [17]	UNCLEAR: Although hospital database was used to identify MDR-TB and XDR-TB admissions the study relied on HCW self-reporting their occupation.	Incidence of TB disease: LOW (However, it is possible that HCWs seeking TB treatment may not have stated their occupation, resulting in underreporting of TB cases and information bias)	UNCLEAR: Underreporting of TB cases; Community vs occupational exposure to TB; HIV status of all of the HCWs not known
Tudor et al. [18]	LOW: Data abstracted from occupational health employee medical charts using a standardized chart audit form	Incidence of TB disease: LOW	UNCLEAR: Underreporting of TB cases; Community vs occupational exposure to TB; HIV status of all of the HCWs not known
Van Rie et al. [23]	UNCLEAR: Participation in the study was voluntary. 120/450 eligible HCWs and 79/296 eligible medical students participated in the study.	Prevalence of LTBI: LOW LTBI was diagnosed using TST and IGRAs	UNCLEAR: Community vs occupational exposure to TB. HIV status of all participants was assessed.
Wilkinson et al. [21]	LOW: Staff TB data was extracted confidentially from the anonymized tuberculosis control programme register.	Incidence of TB disease: LOW (Case ascertainment is known to be complete because tuberculosis treatment cannot be obtained anywhere else in the district and all staff illness episodes are recorded in personnel files)	UNCLEAR: Community vs occupational exposure to TB; HIV status of all of the HCWs not known

*LOW* low risk of bias, *HIGH* high risk of bias *UNCLEAR* unclear risk of bias

^a^Selection bias refers to systematic differences between baseline characteristics of the groups that are compared or characteristics of those who participate in the study and those who don’t. It is important that the group described is representative of the population of interest

^b^Detection bias/information bias refers to systematic differences between groups in how outcomes are determined. Participant’s self-reported outcomes are usually associated with a high risk of detection or information bias

^c^Confounding factors can cause or prevent the outcome of interest, are not intermediate variables, and are not associated with the factor(s) under investigation. Confounding factors result in situations in which the effects of two processes are not separated, or the contribution of causal factors cannot be separated, or the measure of the effect of exposure or risk is distorted because of its association with other factors influencing the outcome of the study

**Table 4:** Risk factors associated with active TB disease or latent TB infection (LTBI) in health care workers

Study ID	Age	Employment duration	Occupation	HIV status	Diabetes	TB IPC training
Incidence of active TB disease						
Ayuk et al. [20]	Not significantly associated with odds of acquiring TB disease; age 40–49 years most affected by TB disease	Not significantly associated with odds of acquiring TB disease	Not assessed	HIV+ HCWs have significantly increased odds of acquiring TB disease (OR: 67.08 95%CI: 7.5–596.6)	Not significantly associated with odds of acquiring TB disease (OR: 1.7 95%CI: 0.8–3.8)	No previous training in TB prevention (OR: 2.97 95%CI: 1.2–7.7); no knowledge of TB risk profile of work place (OR: 8.7 95%CI: 1.1–67.96) significantly associated with increased odds of acquiring TB disease
Tudor et al. [18]	Not significantly associated with incidence of TB disease	Years worked in hospital not significantly associated with incidence of TB disease	No significant association between occupational category and incidence of TB disease; history of working in TB ward significantly associated with increased incidence of TB disease (IRR: 2.87 95 % CI: 1.67–4.93)	HIV-positivity significantly associated with increase incidence of TB disease (adjIRR: 3.2 95%CI: 1.54–6.66)	Not reported on	Not reported on
Incidence of latent TB infection						
Adams et al. [14]	Age 31–40 years significantly associated with increased odds of LTBI (OR: 2.08 95%CI: 1.04, 4.17)	Employment in primary level health care facility > 20 years significantly associated with increased odds of LTBI (OR: 3.47, 95%CI 1.01–11.97)	Not assessed	3/5 HCWs with TB disease were HIV + HIV+ significantly associated with decreased odds of TSTpos (OR: 0.41 95%CI: 0.17–0.95)	Not significantly associated with odds of LTBI	In secondary level staff “some training on self-protection from TB infection” significantly associated with decreased odds of LTBI (OR: 0.38 95%CI: 0.16–0.91)
McCarthy et al. [22]	IGRA: ≥ 31 years significantly associated with increased risk of latent TB infection (crude IRR: 2.3 95%CI: 0.9, 5.8) TST: No significant association	Not reported on	IGRA: HCWs had a significantly greater risk of acquiring latent TB infection (crude IRR: 4.32, 95%CI: 1.7–12.2) TST: No significant association	No significant association between HIV status and risk of latent TB infection with both IGRA and TST	Not reported on	IGRA: Higher TB knowledge score (crude IRR: 0.4, 95%CI: 0.1, 1.3) and TB infection control training (crude IRR: 0.4, 95%CI: 0.1, 1.2) significantly reduced risk of latent TB infection TST; TB infection control practiced by participants significantly reduced risk of latent TB infection (crude IRR: 0.4, 95%CI: 0.1, 1.3)
Prevalence of latent TB infection						
Van Rie [23]	Prevalence of LTBI No significant association with LTBI in medical students or health care workers	Not reported on	Not assessed in medical students No significant association with LTBI in health care workers	Medical students all HIV-negative HIV-negative health care workers significantly reduced odds of TST positive (OR: 0.28 95%CI: 0.1–0.74)	Not reported on	Medical students: TB knowledge score > 7 (median score) significantly decreased odds of LTBI (adjOR: 0.29 95 % CI: 0.09–0.98) Health care workers: no significant association

### Incidence of active TB disease in health care workers

Ten studies reported on the incidence of active TB disease in South African health care workers. Six of the ten studies reporting on the incidence of active TB disease in health care workers show a higher incidence of active TB disease in health care workers compared to either the general population or people in the surrounding community (Table 2) (Dwadwa et al: Health worker access to HIV/TB prevention, treatment and care services in Africa: situational analysis and mapping of routine and current best practices, unpublished) [14–18].

**Fig. 2:**
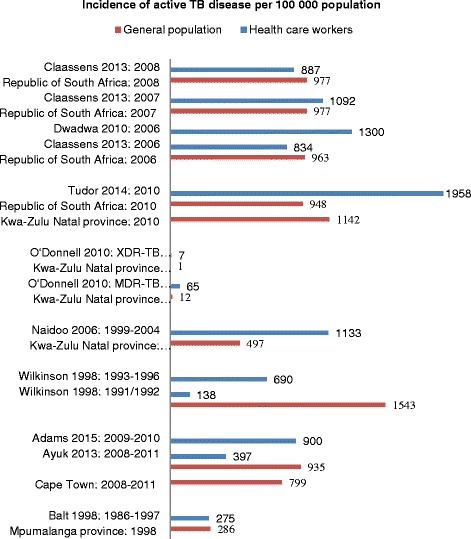
Bar graph comparing incidence of active TB disease in health care workers and general population. Dwadwa et al: Health worker access to HIV/TB prevention, treatment and care services in Africa: situational analysis and mapping of routine and current best practices, unpublished: Data presented is an average of the combined incidence of active TB disease in health care workers from best practice and randomly selected health care facilities; all incidence data for general population in the Republic of South Africa was obtained from the World Health Organisation Global TB database [19]; data on the incidence of active TB disease in the surrounding community was obtained from the individual studies; MDR-TB: multi-drug resistant tuberculosis; XDR-TB: extensively-drug resistant tuberculosis

Both national surveys reported a higher incidence and prevalence of active TB disease in health care workers compared to the general population (Fig. 2). One national survey of 133 primary health care facilities, conducted between 2006 and 2008, reported a 2–3 times higher incidence of active TB disease in health care workers compared to the general population [15] (Table 2). The authors noted that only 40 % of surveyed facilities had a TB screening program for health care workers. Many cases, therefore, are thought to go unreported [15]. The other national survey was a situational analysis conducted in 2006 that investigated health care worker access to HIV/TB prevention, treatment and care services in South Africa. This survey reported an active TB disease incidence of 1.13 to 1.47 % in health care workers from various publicly funded health care facilities (Dwadwa et al: Health worker access to HIV/TB prevention, treatment and care services in Africa: situational analysis and mapping of routine and current best practices, unpublished). In contrast, in 2006 the incidence of active TB disease in the South African population was 963 per 100 000 population (approximately 1 %) [19].

Studies conducted in public sector hospitals in KwaZulu-Natal during the time period 1999 to 2010 found that health care workers in these facilities had significantly increased incidence of both drug sensitive and drug-resistant TB disease compared to the surrounding communities (Fig. 2) [16–18]. In contrast to this, studies conducted in the Western Cape province between 2008 and 2011, showed that health care workers from primary, secondary and tertiary care hospitals had a similar or lower incidence of active TB disease compared to the general population at the city and provincial level [14, 20].

Four studies reported the incidence of active TB disease for the various occupational categories [16, 18, 20, 21]. While certain categories of health care workers appeared to have higher incidence of active TB disease, only one study reported a statistically significant difference in incidence of active TB disease between the various occupational categories [20]. In this study, conducted in a tertiary hospital in the Western Cape, the incidence of active TB disease in the housekeeping staff was three times that of the entire health care workforce (risk ratio: 3, 95%CI: 2.7–3.3) and nurses (risk ratio: 3.7, 95%CI: 3.2–4.1) and six times that of doctors (risk ratio: 6.1, 95%CI: 5.2–7.1) [20]. Furthermore, the incidence of active TB disease was statistically significantly greater in nurses compared to doctors (324/100 000 population vs. 194/100 000 population, risk ratio: 1.7, 95%CI: 1.4–2) [20] (Table 2).

### Prevalence of active TB disease in health care workers

Two studies provide data on the prevalence of active TB disease in South African health care workers. Active TB disease was less prevalent in health care workers working in health care facilities (1.4 %) [14] compared to those working in the community (5 %) [6]. Both of the studies report a higher prevalence of TB disease in health care workers compared to the prevalence in the surrounding community (population-based prevalence survey reported a TB disease prevalence of 3 %[6]) or general population (national prevalence of active TB disease for 2010 was estimated to be 0.8 % [19]) [6, 14].

### Incidence of latent TB infection in health care workers

Two studies provided information on the incidence of latent TB infection in health care workers in South Africa. Health care workers from primary and secondary level health care facilities in the Western Cape province had an annual rate of latent TB infection of 38 % (95%CI: 22–56 %, converted from negative to positive TST during 2009–2011) [14]. In Gauteng province, a cohort of health care workers and medical students working in three public sector health care facilities had an annual incidence of latent TB infection of 27 % (25/93 converted from negative to positive TST during 2008–2009) [22].

### Prevalence of latent TB infection in health care workers

Three studies reported on prevalence of latent TB infection in health care workers in South Africa. The prevalence of latent TB infection in health care workers ranged from 84 % (TST-positive, health care workers from primary and secondary health care facilities in the Western Cape; 2009) [14] to 48 % (TST-positive, medical students and health care workers from public sector health care facilities in Gauteng; 2008) [23]. Two of the three studies reporting on prevalence of latent TB infection were conducted in the Western Cape [14, 24]. In Gauteng, the prevalence of latent TB infection was higher among HCWs than medical students (56.7 % vs. 26.6 %, respectively; *p* < 0.0001) [23].

### Drug-resistant TB in health care workers

Two studies specifically report on drug-resistant TB in health care workers in South Africa. One study reported specifically on the incidence of hospital admissions, to a public referral hospital in KwaZulu-Natal, for multidrug-resistant or extensively drug resistant TB disease among health care workers [17]. In this study the annual estimated incidence of hospital admission for multidrug resistant (MDR)-TB among health care workers was more than 5 times that of the adult general population and more than 6 times of that of general population for extensively drug resistant (XDR)-TB (Table 2). A further retrospective case record review reported that ten of the 334 patients diagnosed with (XDR)-TB between 1996 and 2008 in the Eastern and Western Cape were health care workers [25].

### Risk factors for occupational TB in health care workers

The findings of the five studies investigating the risk factors associated with active TB disease or latent TB infection (LTBI) in health care workers are presented in Table 4.

#### Age of health care worker

There wasn’t a consistent association across the studies with regards to health care worker age and risk of active TB disease or latent TB infection. Furthermore, the particular age category of health care worker most affected by active TB disease or latent TB infection also differed between studies (Table 4) and only two studies reported a significant difference in the incidence of active TB disease between the various age categories. Ayuk et al. [20] reported that health care workers 40 years or older had a significantly higher incidence of active TB disease compared to health care workers younger than 40 years (incidence rate difference: 206/100 000, *p* = 0.038). In Naidoo et al. [16], health care workers aged 25–29 years had a significantly higher incidence of active TB disease compared to all other age groups (mean incidence: 2467/100 000, SD 1184, *p* = 0.01). Of the five studies reporting on this, two studies found a significant association between age and risk for latent TB infection. In Adams et al. [14] health care workers in a particular age group (31–40 years) had a significantly increased odds of acquiring latent TB infection (odds ratio (OR): 2.08 95%CI: 1.04, 4.17). McCarthy et al. [22] found that health care workers 31 years and older had a significantly higher risk of acquiring latent TB infection (measured by IGRA; crude IRR: 2.3 95%CI: 0.9, 5.8).

#### Duration of employment

Both of the studies that assessed the relationship between duration of employment and incidence of active TB disease found no significant association [18, 20]. In the one study that assessed the relationship between duration of employment and incidence of latent TB infection, duration of employment of greater than 20 years in a primary level care facilities was statistically significantly associated with an increased risk of latent TB infection (TST positivity; OR: 3.47; 95 % CI: 1.01–11.97) [14].

#### Health care worker occupation and work location

One study assessed the relationship between health care worker occupation and incidence of active TB disease and found no significant association for the various occupation categories [18]. No significant association was found between health care worker job category and risk of latent TB infection in the one study that reported on this relationship [23].

Tudor et al. [18] reported that compared to health care workers with no history of working in these areas, health care workers with a history of working in the TB ward (IRR 2.03, 95 % CI 1.11–3.71), paediatric ward (IRR 1.82, 95 % CI 1.07–3.10), the outpatient department (IRR 2.08, 95 % CI 1.23–3.52) and the stores department (IRR 2.38, 95 % CI 1.06–5.34) had a significantly increased incidence of active TB disease.

#### HIV status

Two studies reported on the relationship between HIV status and the incidence of active TB disease [18, 20]. Health care workers living with HIV had significantly greater odds of developing TB (Tudor et al. [18]: adjusted OR 4.11; 95 % CI: 1.95–8.67; Ayuk et al.: adjusted OR = 50.94; adjusted 95 % CI: 5.26–493.73, *p* = 0.003) [18, 20].

Two studies assessed the relationship between HIV status and incidence of latent TB infection [14, 22]. Adams et al. [14] showed that HIV-positive health care workers were less likely to have a positive TST (OR: 0.41, 95 %CI: 0.18–0.92). In contrast, McCarthy et al. [22] did not find a significant association between HIV status and incidence of latent TB infection as measured by either IGRA assay or TST.

Only one study reported on the association between HIV status and the prevalence of latent TB infection [23]. Although HIV-negative health care workers were less likely to have a positive TST (OR 0.28; 95 % CI 0.10–0.74), this association was not significant after adjustment for age, job category and TB knowledge score [23].

In KwaZulu-Natal, Wilkinson et al. [21] reported that 12 out of the 14 TB cases in health care workers tested for HIV were positive. O’Donnell et al. [17] reported that of those tested, 67 % of the health care workers admitted to hospital for MDR-TB or XDR-TB were HIV-positive. Tudor et al. [18] reported that 21 % of the health care workers with TB were HIV-positive. In the Western Cape, Adams et al. [14] reported that 3/5 health care workers newly diagnosed with TB were HIV-positive.

#### Diabetes

There is limited data from the studies in this review on the association between diabetes and risk of TB disease or infection. Both of the nurses diagnosed with TB in Balt et al. had non-insulin dependent diabetes [26]. However, no significant association between diabetes and risk of TB disease or infection was noted in the two studies reporting on this relationship [20] (Adams: Prevalence and determinants of TB infection in health care workers, unpublished).

#### TB infection prevention and control (IPC) training

One study assessed the association between training in TB IPC and risk of active TB disease and found that health care workers who reported having had no previous TB IPC training were significantly more likely to acquire active TB disease (OR: 2.97, 95%CI: 1.15–7.71) [20]. Two studies reported on the association between TB IPC knowledge, training and practice and the risk of incident latent TB infection [14, 22]. Adams et al. [14] found that having “some training in infection control procedures aimed at self-protection” significantly reduced the odds of latent TB infection (TST positivity) in health care workers at secondary level health care facilities (OR = 0.38; 95 % CI: 0.17–0.87). Similarly, McCarthy et al. [22] found that a higher TB knowledge score (as measured by IGRA, crude IRR: 0.4, 95%CI: 0.1, 1.3), TB infection control training (as measured by IGRA, crude IRR: 0.4, 95%CI: 0.1, 1.2) and TB infection control practiced by participants (as measured by TST, crude IRR: 0.4, 95%CI: 0.1, 1.3) significantly reduced the risk of incident latent TB infection.

Van Rie et al. [23] found that medical students who had a TB knowledge score > 7 (the median score for all participants), had reduced odds of a positive TST by >70 % (adjusted OR: 0.29; 95 % CI: 0.09–0.98).

## Discussion

The majority of the studies reflect a high burden of active TB disease and latent TB infection in health care workers in South Africa. Although ten studies provide information on the incidence of active TB disease, only two studies provide information on the prevalence of active TB disease in health care workers in South Africa. Furthermore, only two studies reported on the incidence of latent TB infection with only three studies reporting on the prevalence of latent TB infection. Only two studies reported on the burden of specifically drug-resistant TB in South African health care workers. Most of the studies were conducted in the provinces of Kwa-Zulu Natal and the Western Cape, with little to no information on the burden of active TB disease or latent TB infection in health care workers employed in health care facilities in the Eastern Cape, the Free State, the Northern Cape and the North West province. Considering the importance of the issue it is surprising that there is relatively little research describing the actual burden of TB in health care workers in South Africa.

Other reviews have shown that the local prevalence of TB and HIV influences the risk and burden of active TB disease in health care workers in those localities [1–3]. The background incidence of active TB disease differs between provinces in South Africa, with the background incidence/prevalence of active TB disease in KwaZulu-Natal being one of the highest in the country. This may explain why the incidence of active TB disease was consistently higher in health care workers compared to the community in Kwa-Zulu Natal province and not in the Western Cape province.

Previous reviews have shown that the infectiousness of the patient population and the level of care of the facility, influence the extent of TB transmission to health care workers [1–3]. Health care workers at primary care facilities and those working in the community may be more likely to be exposed to infectious, as yet undiagnosed, TB patients compared to health care workers at tertiary care facilities. The studies reporting on prevalence of active TB disease, conducted in the Western Cape at a similar time (2009/2010), support this in that active TB disease was more prevalent in community health workers (5 %) [6] compared to health care workers in primary and secondary health care facilities (1.4 %) (Adams: Prevalence and determinants of TB infection in health care workers, unpublished).

Two studies provide information on the incidence of latent TB infection in health care workers. In both cases the annual incidence of latent TB infection in these South African health care workers is three to four times higher than the incidence previously reported in health care workers in India, another high TB incidence setting [14, 27, 28].

There is limited data on the various risk factors for acquisition of active TB disease or latent TB infection in health care workers in South Africa (Table 3). HIV status and training in TB infection, prevention and control were the only two factors for which there were consistent associations. While HIV-positivity was significantly associated with increased odds of acquiring active TB disease, it was not consistently associated with increased odds of acquiring latent TB infection. The treatment of HIV and latent TB infection in health care workers will need to be explored in future studies to understand the impact on the development of active TB disease in HIV-infected health care workers.

In four studies training in TB infection prevention and control was significantly associated with decreased odds of acquiring active TB disease [20] and latent TB infection [14, 22, 23]. This finding emphasizes the importance of on-going TB infection prevention and control training among all health care workers in the facility. Considering these findings and that all employees of the health care facility are at risk of acquiring TB it can be emphasized that all employees of the health care facility should receive training in TB infection, prevention and control.

Previous reviews reported that the specific work locations and occupational categories of health care workers are associated with a higher risk of acquiring TB disease [1–3]. The studies in our review that investigated these relationships showed somewhat similar findings. In one study there was no significant association between health care worker occupation and incidence of active TB disease, however, specific work locations (TB ward, paediatric ward and the outpatient department) were significantly associated with increased risk of acquiring active TB disease [18]. In the other studies certain occupational categories of health care workers appeared to have higher incidence of active TB disease, however, only one of the four studies reporting on this found the difference in incidence rates between the various occupational categories to be statistically significantly different [20]. Nevertheless, it was startling to see the high incidence of active TB disease in housekeeping staff, paramedical staff and hospital security staff [16, 20]. Housekeeping and hospital security staffs, in particular, are unlikely to receive training in appropriate TB infection prevention and control, providing a possible explanation for the high burden of TB in these individuals.

## Conclusions

There is relatively little research on the epidemiology of TB in health care workers in South Africa, despite the importance of the issue. Only two studies provided information on the annual incidence of latent TB infection in health care workers in South Africa. In addition to this, there is a dearth of information on the epidemiology of TB in health care workers from Eastern Cape, Free State, the Northern Cape and the North West provinces of South Africa. The lack of comprehensive information on the true burden of TB in health care workers will have implications on the implementation of preventive therapy policies.

To determine the true extent of the TB epidemic in health care workers in South Africa, screening for active TB disease (with identification of drug resistant TB strains) should be conducted in all health care facility employees, in all health care facilities on a regular basis. Future research should investigate the optimal approach, in terms of cost-effectiveness, feasibility, practicality and usefulness, to TB screening for active TB disease in health care workers in South Africa.

Finally, the evidence base shows a high burden of both active and latent TB in health care workers in South Africa, necessitating an urgent need to improve existing TB infection, prevention and control measures in South African health care facilities. Further to this training in TB infection, prevention and control be provided to all health care facility employees, including non-clinical staff.

## Abbreviations

HCW, health care worker; IGRA, interferon-gamma release assays; IRR, incident rate ratio; LTBI, latent tuberculosis infection; MDR-TB, multi-drug resistant tuberculosis; OR, odds ratio; TB, tuberculosis; TST, tuberculin skin test; XDR-TB, extensively drug-resistant tuberculosis.

## Additional file

Additional file 1: Table S1. List of excluded studies with reasons for exclusion. (DOCX 86 kb)
