# Supplementary material for: The epidemiology of tuberculosis in health care workers in South Africa: a systematic review
Source: BMC Health Serv Res. 2016 Aug 20;16:416. doi: 10.1186/s12913-016-1601-5 (PMC4992336; doi:10.1186/s12913-016-1601-5)
Supplement: Additional file 1: Table S1. — List of excluded studies with reasons for exclusion. (DOCX 86 kb) [file 12913_2016_1601_MOESM1_ESM.docx]

Table 1: List of excluded studies with reasons for exclusion

| **Study ID** | **Reason for exclusion** |
| --- | --- |
| Baussano 2011 | Review |
| Bateman 2013 | Letter |
| Bock 2007 | Review article |
| Claassens 2010 | Community-based health care researchers |
| Cooke 2011 | Case study |
| Cullinan 2002 | News/letter |
| Detjen 2009 | No epidemiology or programmatic data provided |
| Dharmadhikari 2012 | Study participants not health care workers |
| Dheda 2014 | Review |
| Dludla 2012 | Letter |
| Fairall 2005 | No epidemiology or programmatic data provided |
| Forder 2007 | Review/commentary general Infection Control |
| Ghandi 2013 | Participants not health care workers |
| Goemere 2007 | Letter |
| Gonzalez-Angulo 2013 | Study participants not health care workers |
| Harries 1997 | Review |
| Heunis 2011 | Qualitative research on accuracy of TB data |
| Harrop 2011 | Review |
| Joshi 2006 | Review |
| Kleinert 2009 | Review article/commentary |
| Koenig 2008 | Letter/Commentary |
| Lewin 2005 | Aimed at TB treatment adherence for all participants |
| Menzies 2007 | Review |
| Metah 2008 | Review article/commentary |
| Motosomane 2008 | Qualitative research |
| Nathanson 2010 | Review article/commentary |
| Naidoo 2010 | Review on IC |
| Padayatchi 2010 | No epidemiology or programmatic data provided |
| Rowe 2005 | Study participants are not health care workers |
| Shenoi 2010 | Review article |
| Shenoi 2013 | TB case-finding by “cough officer” in clinic patients |
| Shisana 2004 | Prevalence of HIV, not TB, in health care workers |
| Sissolak 2010 | TB patients potential to transmit TB |
| Uwimana 2012 | Study participants are not health care workers |
| Yassi 2011 | Overview of infection control project (methodology) |
| Yassi 2012 | Review |
| Zwarenstein 2011 | No epidemiology or programmatic data provided |
